# Genomic characterization of a newly established esophageal squamous cell carcinoma cell line from China and published esophageal squamous cell carcinoma cell lines

**DOI:** 10.1186/s12935-020-01268-x

**Published:** 2020-05-24

**Authors:** Xiang Li, Dongping Tian, Yi Guo, Shiyue Qiu, Zexin Xu, Wen Deng, Min Su

**Affiliations:** 1 Institute of Clinical Pathology, Guangodng Provincial Key Laboratory of Infectious Disease and Molecular Immunopathology, Shantou University Medical College, No. 22 Xinling Road, Shantou, Guangdong Province 515041 People’s Republic of China; 2 The Judicial Critical Center, Shantou University Medical College, Shantou University Medical College, No. 22 Xinling Road, Shantou, Guangdong Province 515041 People’s Republic of China; 3 Cancer Hospital of Shantou University Medical College, No. 7 Raoping Road, Shantou, Guangdong Province 515041 People’s Republic of China; 4 School of Nursing, Li Ka Shing Faculty of Medicine, The University of Hong Kong, 3/F, William MW Mong Block 21 Sassoon Road, Pokfulam, Hong Kong SAR, China

**Keywords:** Esophageal squamous cell carcinoma, Cell line establishment, Karyotype, Mutation signature, Whole genome sequencing

## Abstract

**Background:**

Esophageal squamous cell carcinoma (ESCC) is one of the most prevalent malignancies and a major cause of cancer related death worldwide, especially in China. Cell lines are widely used disease models for basic medical research, however, well characterized ESCC cell models from China were seldom reported. Misidentifying and cross-contaminations of cell lines also hamper the way of producing solid and reproductive data.

**Methods:**

CSEC216 was originated from a 45-year-old male ESCC patient from Chaoshan littoral, China. Specimens were minced into fragments and seeded in T-25 flask for primary culture. Immunoflourescence staining was performed for identifying the origination and proliferation activity. In vitro migration and invasion abilities was tested by transwell assay. DNA Short Tandem Repeats profiling was implemented for cell authorization. Karyotype was investigated by spectrum karyotyping. Whole genome sequencing was utilized to investigate genomic alterations. Background information and genomic mutation data of published ESCC cell lines were obtained from online databases.

**Results:**

CSEC216 was an uncontaminated cell line, exhibited epithelial cell features with polygonal morphology and adherent growth as monolayer. Immuno staining demonstrated its epithelial origination and high proliferation rate. The Population Doubling time was 29.7 h. The karyotype demonstrated tumor cell patterns with aneuploidy and complex chromosomal aberrations. Mutation signatures, genes with SNA or CNA of CSEC216 and published ESCC cell lines were similar with the mutation spectrum of original ESCC tumors.

**Conclusions:**

ESCC cell line CSEC216 from high incidence region in China was established with no cross-contamination. Biological features were studied. Genomic mutation features of CSEC216 and 28 ESCC cell lines were characterized which provided thorough cytogenetic background that facilitated future usage.

## Background

Esophageal cancer (EC) is the eleventh most commonly diagnosed malignancy, and the sixth leading cause of cancer mortality worldwide, an estimated number of 483,000 new cases and 439,000 deaths occurred in 2015 worldwide [1]. In China, EC ranks third in incidence and fourth in mortality [2]. But the incidence of EC varies geographically, with the Chaoshan littoral possessing one of the highest incidence rate of 74.47 per 100,000, comparing worldwide incidence of 5.2 per 100,000 [3, 4], Though progress has been made in diagnosis and treatment, the prognosis of EC patients remains poor and effective treatment remains challenging.

Sustainable and uncontaminated cancer cell lines provide available resources for researchers to utilize. Human cell lines are now widely used in laboratories worldwide. The first continuous cell line Hela, was established in 1952 (7). In 1981, Nelson-Rees et al. reported that many cell lines had been switched or cross-contaminated with Hela cells. To date, a significant proportion of cell based research is misleading and misrepresentative, because the cell lines are of different origin to that claimed or the cross-contamination between cell lines [5]. The number of published cases of cross-contamination is still increasing [6]. Therefore, authorization of newly established cell lines is utterly needed.

There are abundant esophageal squamous cell carcinoma (ESCC) cell lines around the world. However, few of them were derived from China. Here we report a newly established and authorized ESCC cell line designated CSEC216, derived from a 45-year old male patient from the Chaoshan littoral. This cell line would serve as potent in vitro model for the exploration of the pathogenesis of ESCC. The current study also reported the genomic similarity of 28 ESCC cell lines and original ESCC tumors.

## Materials and methods

### Patient background

**Fig. 1:**
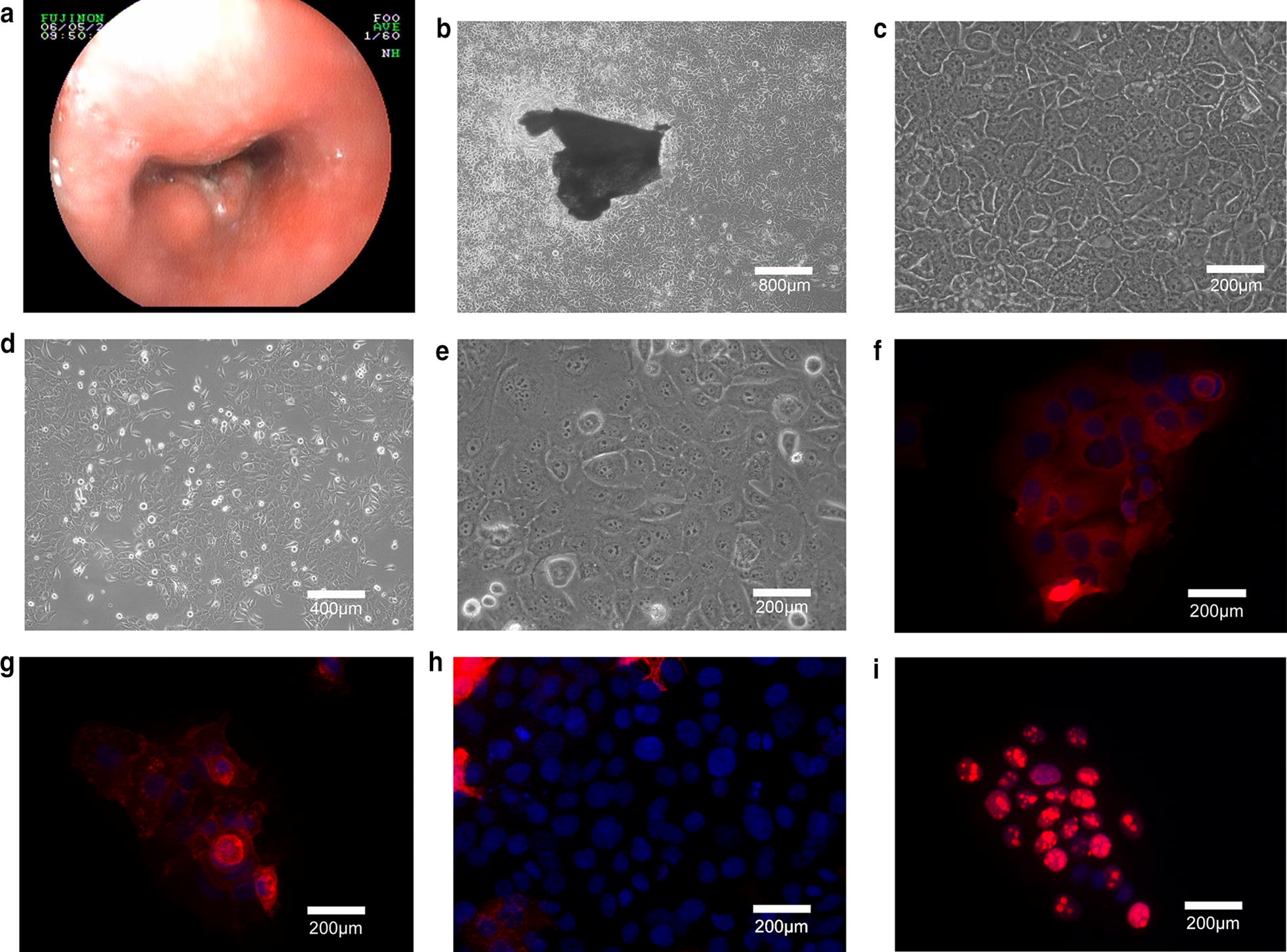
Endoscopic image of original tumor, morphological and immunofluorescence staining photos of derived cancer cells. **a** Endoscopic photograph of the original tumor. **b**–**e**, Morphology of CSEC216 cells at P0. **f**, Cytokeratin positively expressed on cytomembrane with red color. **g**, **e**-cadherin positively expressed on cytomembrane with red color. **h** Vimentin negatively expressed. **i** Ki-67 positively expressed in nuclear with red color

CSEC216 was established from an endoscopic biopsy sample derived from a 45-year-old male patient who underwent esophageal endoscopy in the Cancer Hospital of Shantou University Medical College. The tumor located at middle thoracic esophagus, 23–30 cm from the incisor with constrictive lumen and elevated mucosa observed in the esophageal tract (Fig. 1a).

Written informed consent was received from the patient.

### Primary culture

The endoscopic specimen was immersed in a 5 ml tube filled with culture medium (Dulbecco’s modified Eagle’s medium with 10% fetal bovine serum, 100 U/ml penicillin, 100 mg/ml streptomycin). The specimen was then washed 3 times in PBS and minced with a sterile scalpel into small fragments of 1 mm^3^. The tissue fragments were placed in a collagen coated T-25 flask containing 2 ml culture medium and gently placed in a 37 °C incubator containing a humidified atmosphere and 5% CO_2_. Cells were grown for 7 days. After which the medium was replaced every 3 days. Stromal fibroblasts were eliminated by differential trypsinization [7]. Cells were suspended with 0.25% trypsin and 0.02% EDTA treatment and propagated at a split ratio of 1:2 or 1:3 depending on their growth rate. The cells were resuspended in freezing medium (90% FBS + 10%DMSO) and stored in liquid nitrogen from passage 2.

### Growth characteristics

The CSEC216 cell line had been subcultured for 50 passages, during which the total culturing time and population doubling time(PDT)^1^ were used for growth curve plotting.

For population doubling time calculation, 10,000 cells at passage 28 in 300 μl culture medium were inoculated into each well of a 24-well plate and culture medium was replaced every 3 days. Three wells of cells were harvested by trypsinization each time with 24 h intervals, the cell number of each well was determined using a Neubauer hemacytometer, then the cell numbers were averaged. Cell growth during log phase was used to calculate doubling time (Roth V. 2006 Doubling Time Computing, http://www.doubling-time.com/compute.php 18/10/2015).

1 $$ \begin{aligned} DoublingTime = \frac{Duration \times \log \left( 2 \right)}{{\log \frac{FinalConcentration}{InnitialConcentration}}}. \hfill \\ \hfill \\ \end{aligned} $$

### Karyotypic analysis

The karyotype of CSEC216 cells was analyzed by spectral karyotyping (SKY). To obtain cells at metaphase, cells at 70% confluence at passage 20 were treated with 0.03 μg/ml colcemid for 7 h then harvested by trypsinization. After neutralization by mixing the cell suspension with equal amount of culture medium and centrifugation at 1000 rpm, the supernatant was discarded. Cells were resuspended in 2 ml 0.8% sodium citrate and kept at 37 °C for 15 min. 250 μl fresh made fix solution (methanol:acetic acid = 3:1) was added to the cell suspension at 37 °C for 5 min to prefix the cells. Cells were collected by centrifugation and washed 3 times with fix solution. After the final wash, supernatant was discarded. Cells were resuspended in 500 μl fix solution and 15 μl cell suspension was dropped onto an adhesive slide, air dried and the hybridization area was marked with a diamond pen. The slide was stored at room temperature and kept from light for 7 days. SKY was performed using a SKY probe kit SkyPaint™ mixture (Applied Spectral Imaging, Migdal a’Emek, Israel), according to the manufacturer’s instruction). The karyotype was analysed by SkyView™ Imaging System (Applied Spectral Imaging) equipped with a Zeiss Axioplan-2 fluorescence microscope.

### Whole genome sequencing (WGS)

#### Library preparation for sequencing

A total amount of 0.5 μg DNA per sample was used for the DNA library preparations. Sequencing library was generated using Truseq Nano DNA HT Sample Prep Kit (Illumina USA) following manufacturer’s recommendations and index codes were added to each sample. Briefly, genomic DNA sample was fragmented by sonication to a size of 350 bp. Then DNA fragments were end-polished, A-tailed, and ligated with the full-length adapter for Illumina sequencing, followed by further PCR amplification. After PCR products were purified (AMPure XP system), libraries were analyzed for size distribution by Agilent 2100 Bioanalyzer and quantified by real-time PCR (3 nM).

The clustering of the index-coded samples was performed on a cBot Cluster Generation System using HiSeq X PE Cluster Kit V2.5 (Illumina) according to the manufacturer’s instructions. After cluster generation, the DNA libraries were sequenced on Illumina HiSeq platform and 150 bp paired-end reads were generated.

#### Quality control

Raw data (short reads) in FASTQ format (.fq) were processed though in–house perl scripts. In this step, cleans data (clean reads) were obtained by removing reads containing adapter, reads containing poly-N and low quality reads from raw data. At the same time, Q20, Q30, error rate and GC content of clean data were calculated. All the further analysis was based on the high quality clean data.

#### Analysis

Mapping: Burrows-Wheeler Aligner (BWA2) software is utilized to map the paired-end clean reads to the reference genome (UCSC hg19). The original mapping result in BAM format can be obtained. Picard, GATK and Samtools are then used to do duplicate removal, local realignment, and base quality recalibration etc.

Single Nucleotide Alterations (SNA) GATK HaplotypeCalleris used to do variant calling and identify SNA, insertions and deletions (INDELS). In this step, raw callsets with false positive variant calls are obtained. Then variant Filtration in GATK is performed to make the raw callsets suitable for meaningful analysis. High quality mutations were retained for germline mutations filtration. Mutations identified in gnomad database (hg19_gnomad211_genome, http://annovar.openbioinformatics.org/en/latest/user-guide/download/) were regarded as germline mutations. Somatic SNA data were utilized for mutational signature analysis by R package MutationalPatterns to decypher the probable mechanism underlying the mutational process. COSMIC cancer mutation signatures were used as reference signatures [8, 9].

Copy Number Alteratons(CNA) Control-FREEC was utilized to do somatic CNA detection. GISTIC algorithm was used to infer recurrently amplified or deleted genomic regions. G-scores were calculated for genomic and gene-coding regions on the basis of the frequency and amplitude of amplification or deletion affecting each gene. A significant CNA region was defined as having amplification or deletion with a G-score > 0.1, corresponding to a p-value threshold of 0.05 from the permutation-derived null distribution.

#### Immunofluorescence (IF) staining

IF staining against Cytokeratin (ready to use, ZSGB-BIO), E-Cadherin (ready to use, ZSGB-BIO),Vimentin (ready to use, ZSGB-BIO), and Ki-67 (ready to use, ZSGB-BIO) was performed to confirm the origination and proliferation activity of CSEC216 cells. Cells were harvested when reached 80% confluence. Then 10,000 cells were inoculated onto a coverslip in a 24-well plate and incubated for 24 h. Cells were fixed with 4% paraformaldehyde at 4 °C for 15 min, permeabilized with 0.5% Triton X-100 at room temperature for 30 min and washed with PBS after each treatment. Primary antibody was applied to the slide and incubated in humidified box at 4 °C overnight. Secondary antibody (Cy3-labeled, Beyotime) was applied after PBS washing and incubated in a humidified chamber at 37 °C for 30 min. The slide was washed with PBS after incubation and mounted by anti-fade DAPI (Prolong Gold antifade reagent with DAPI, Life). Photos were taken by Zeiss Axio CSan Z1.

### Cell line authentication

Genomic DNA of cells at passage 5 was extracted using DNA Mini Kit (QIAGEN) under manufacture’s protocol. DNA samples were sent to Genetica DNA Laboratories USA for Short Tandem Repeats(STR) profiling test. Fifteen STR loci and AMEL were tested. STR electropherogram and signed STR profiling report were shown in supplementary data. Eight STR loci and AMEL were used for online matching in the STR databases of ATCC (https://www.atcc.org/STR_Database.aspx 20/11/2015) and DSMZ (https://www.dsmz.de/fp/cgi-bin/str.html 20/11/2015). Matching criteria for human cell line authentication was set as 0.8 recommended by the website.

### Migration and invasion in vitro analysis

For the in vitro migration assay, 10,000 cells of CSEC216 and KYSE520 [10] were resuspended in Millipore chamber (Hanging Cell Culture Inserts 8 μm PET, Millipore). The chambers were placed in 24-well plate containing 750 μl full complete culture medium and incubated for 24 h. After incubation, non-invaded cells on top of the membrane were scraped off with cotton swab. The chamber was treated with fixing/staining solution (mixture of 4% paraformaldehyde and crystal violet) at room temperature for 30 min.

For invasion assay, the same procedure as in the migration assay was followed. The only difference was that cells were suspended in a mixture of 100 μl FBS free DMEM medium and 100 μl matrigel (BD Biosciences).

### ESCC cell lines from online database

ESCC cell lines were searched on Cellosaurus (https://web.expasy.org/cellosaurus/) [11]. Keyword C4024 (NCI Thesaurus Code for ESCC) was used for searching. A list of problematic ESCC cell lines were downloaded from Cellosaurus database, all ESCC cell line data was shown in Additional file 1: Table S1. CCLE (Cancer Cell Line Encyclopedia) is a database that contains multi-omic data of over a thousand cancer cell lines, 28 ESCC cell lines included. Before took into analysis in this paper, cell lines from CCLE were checked if any problematic cell lines existed. CCLE ESCC cell lines’ background information was shown in Additional file 2: Table S2.

## Results

### Biological properties of CSEC216 cells

CSEC216 cells showed polygonal epithelial cell morphology and adherent growth as monolayer(Fig. 1b–e). Immunofluorescence staining revealed CSEC216 cells positively expressed Cytokine and E-Cadherin but negative for vimentin and the Ki-67 labeling index (LI) was 67.1% (Fig. 1f–i). These results demonstrated that CSEC216 cells were derived from epithelial cells and possessed high proliferation activity. The growth curve demonstrated that CSEC216 cells started to proliferate rapidly after the first subculture and the growth rate becomes steady after the 5th subculture (data not shown). The PDT was 29.7 h at log phase. For migration and invasion in vitro analysis, only minority cells had migrated through the membrane, the migration rate and invasion rate is 3.48% and 1.41% respectively compared with 6.05% and 2.91% of KYSE520 cell line (photo not shown).

### Cell line authentication

In total 15 STR loci and AMEL were analyzed in an STR profiling test. AMEL, D5S818, D13S17, D7S820, D16S539, vWA, TH01, TP0X, CSF1PO STR loci were matched with the STR database of ATCC and DSMZ. No cell lines were matched in ATCC STR database. Cell lines with similar STR loci were matched in DSMZ STR database (data not shown). None of these cell lines was cultured during the CSEC216 establishment. Both results showed that CSEC216 was a unique cell line that was not misidentified and contaminated with other registered cell lines. Full report and electropherogram were shown in Additional file 3: Figure S1.

### Chromosome aberrations

**Fig. 2:**
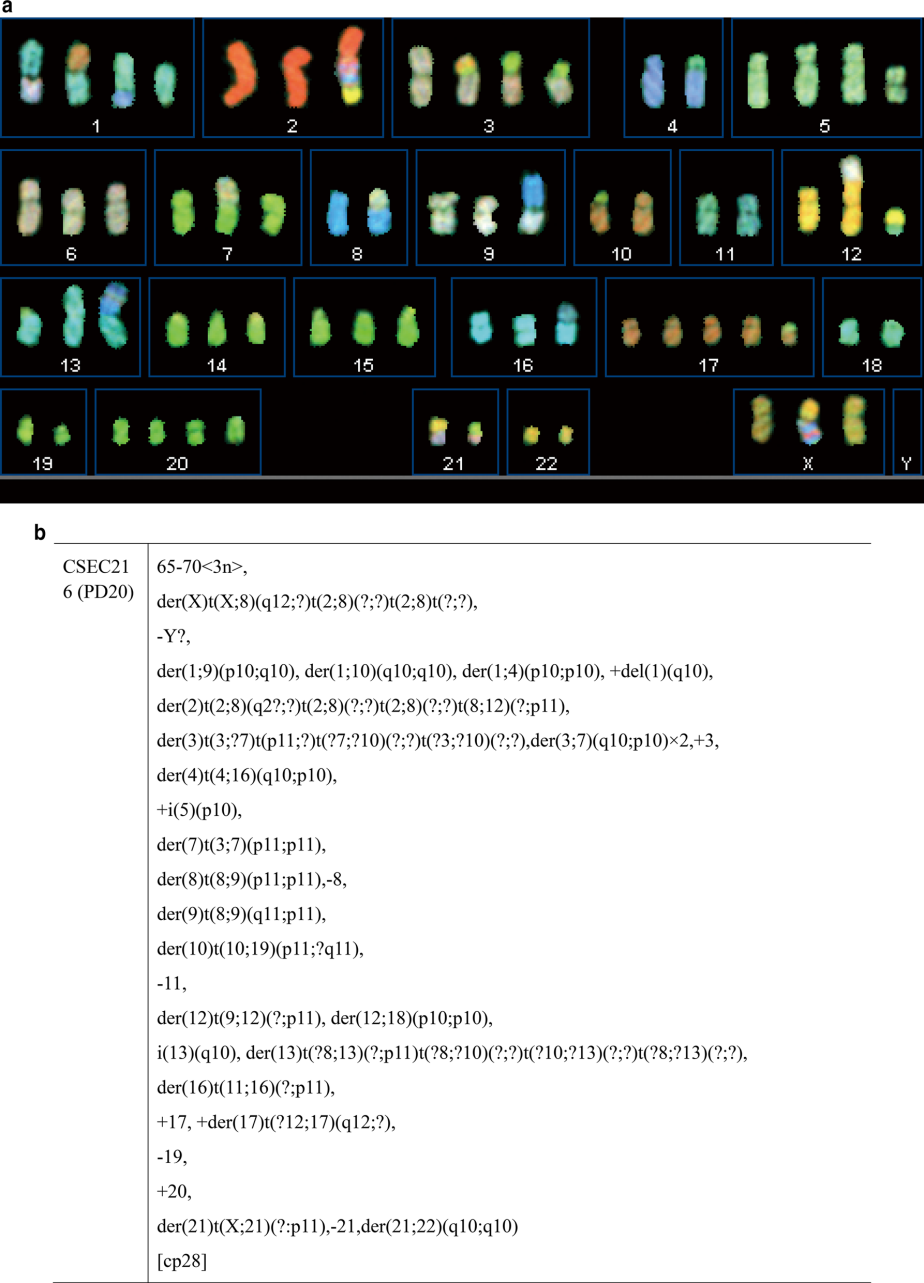
Representative spectral karyotyping photo and report of CSEC216 at passage 20. **a** Metaphase spread chromosomes of CSEC216 labelled with chromosomal specific probes for spectral karyotyping. **b** Summarized karyotypic report of CSEC216 at P20

SKY analysis yielded representative karyotype photograph of CSEC216 cells at passage 20, which was demonstrated in Fig. 2a, karyotypic report was summarized in Fig. 2b. Main clone was hypotriploidy. The chromosome number ranged from 65 to 70. Numerical abnormalities and structural aberrations were common in CSEC216 cells.

Frequently observed chromosomal numerical abnormalities were presented by chromosome loose of Y,8,11,19 and chromosome gain of 1,3,5,17,20. Structural aberrations were complicated as deletion, translocation, isochromosome and derivation chromosome were frequently observed. Derivation chromosome of 2, 3, 13 composed by the most sophisticated translocations was observed, i.e. der (2) t (2, 8) (q2?;?)t(2;8)(?;?)t(2;8)(?;?)t(8;12)(?;p11)、der(3)t(3;?7)t(p11;?)t(?7;?10)(?;?)t(?3;?10)(?;?)、der (13) t(?8;13)(?;p11)t(?8;?10)(?;?)t(?10;?13)(?;?)t(?8;?13)(?;?). Chromosomal break points at p1 or q1 was common in chromosome 1, 2, 3, 4, 5, 7, 8, 9, 10, 12, 13, 16, 17, 18, 21, X.

### Genomic mutations

**Fig. 3:**
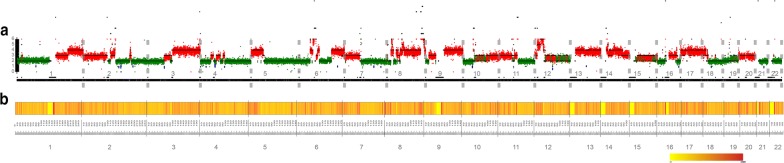
Landscape of CNAs and the density of SNAs and INDELs per megabase along euchromosomes. **a** Landscape of copy number alterations in euchromosomes. **b** The density of single nucleotide alterations, small insertion and deletions per megabase along the chromosomes

**Fig. 4:**
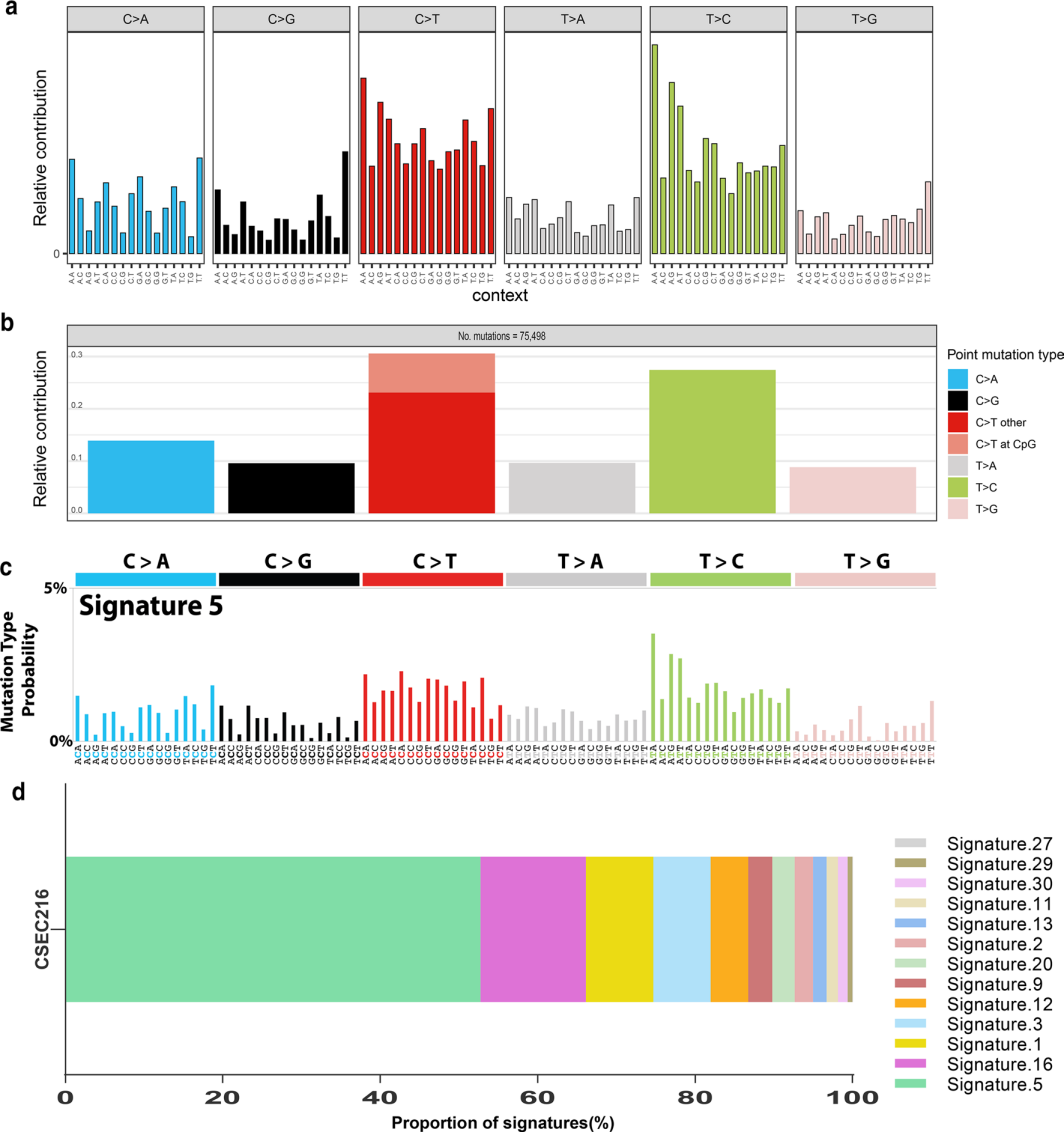
SNA spectrum and mutational signatures of CSEC216. **a** Distribution of 96 trinucleodide mutations spectrum. **b** Relative contribution of mutation type in the 6 base substitution catalogs demonstrated by mutation spectrum. **c** 96 trinucleodide mutations spectrum of COSMIC Signature.5. **d** Optimal contribution of the COSMIC signatures to CSEC216 mutation profile

The genomic DNA of CSEC216 were sequenced with 30X average depth. WGS yielded 3,290,751 SNAs, 572,345 INDELs and 1860 CNA regions. After removal of germline mutations by comparing with gnomad database file (hg19_gnomad211_genome),75527 SNAs and 61866 INDELs were obtained. The genome-wide mutation rate of SNA and INDEL were 2.52 and 2.06 mutations per megabase respectively. The density of SNAs and INDELs per megabase along the chromosomes were plotted in Fig. 3b. C:G > T:A transitions were predominant substitutions which was consistent with our previous work and other published data, accounted for 30.58% (23090/75498) of all SNAs (Fig. 4a, b) [12, 13]. Landscape of CNAs in euchromosomes were showed in Fig. 3a. Genome loss or gain were detected in every chromosome, the size of DNA fragments ranged from 1 KB to 53 MB. Large scale (> 100 kb) amplifications at every chromosomes and large scale deletions at 1p, 2q, 3p, 4p, 4q, 5q, 6q, 7q, 10p, 12q, 13q, 16q, 18q, 20q, 21q, Yq were identified. The frequent copy number changes were elsewise reflected the sophisticated genotype. For copy number gain, the highest copy number was 13 while the lowest was 2. SNA and CNA data of CSEC216 were shown in Additional file 4: Table S3, Additional file 5: Table S4.

Somatic SNAs were converted into a matrix of 96 representative mutations in trinucleotide context for mutation signature analysis (Fig. 4a,b). Mutational signature analysis revealed that the mutational spectrum of CSEC216 were most similar to COSMIC Cancer Signature.5 (COSMIC Mutational Signatures, https://cancer.sanger.ac.uk/cosmic/signatures_v2), which featured by transcriptional strand bias for T > C substitution at ApTpX context, and has been identified in all kinds of cancer types with yet unknown aetiology (Fig. 4c, d).

**Fig. 5:**
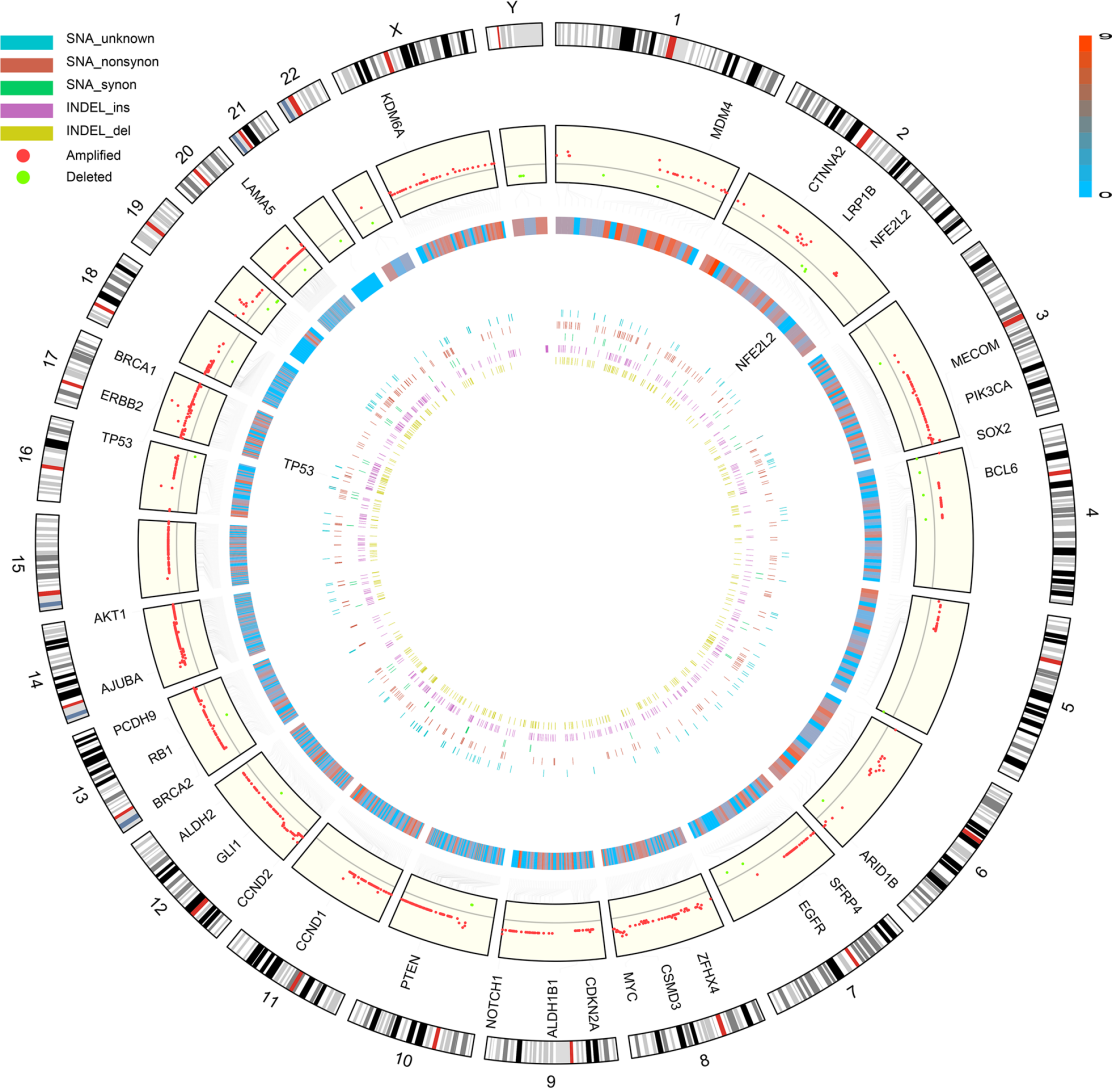
Overview of somatic mutations at exon, up/down stream of CSEC216 genome. Outer track shows the chromosomal ideogram. The second track demonstrate the copy number alterations at exonic regions and labeled with ESCC related genes that were affected. The third track exhibited the log2 converted number of genes within copy number altered regions. The forth track displayed the density of single nucleotide alterations, small insertion and deletions at exonic and up/down stream regions per megabase, labeled with ESCC related genes that were affected

The etiological implication of the mutation signature extracted from all somatic SNAs of CSEC216 was vague. Since 98.8% of somatic SNAs of CSEC216 were located in intron and intergenic region, the mutational context of those SNAs could over rule the mutations that literally contributed to relative etiology. To investigate the impact of genomic mutations to those genes which were implicated to play a role in the carcinogenesis of ESCC, mutations at exon, up/down stream were extracted from SNA, INDEL and CNA data. Resulted in 910 SNAs and 1310 INDELs which affected 999 genes and 14072 genes in 1166 CNA regions. A list of 44 genes related with ESCC were selected from literatures and COSMIC database (Cancer Gene Census, https://cancer.sanger.ac.uk/cosmic/download), of which most genes were chosen from our previous work [12, 14–17]. Gene list were shown in Additional file 6: Table S5. ESCC related genes were mostly found with amplified gene copy number, i.e. TP53, SOX2, EGFR, MYC, CCND1, CDKN2A, NOTCH1, BRCA1, NFE2L2, etc. While only TP53 and NFE2L2 were found in SNAs (Fig. 5). Integrated mutation data of CSEC216 was shown in appendices.

### Genomic characterization of ESCC cell lines

**Fig. 6:**
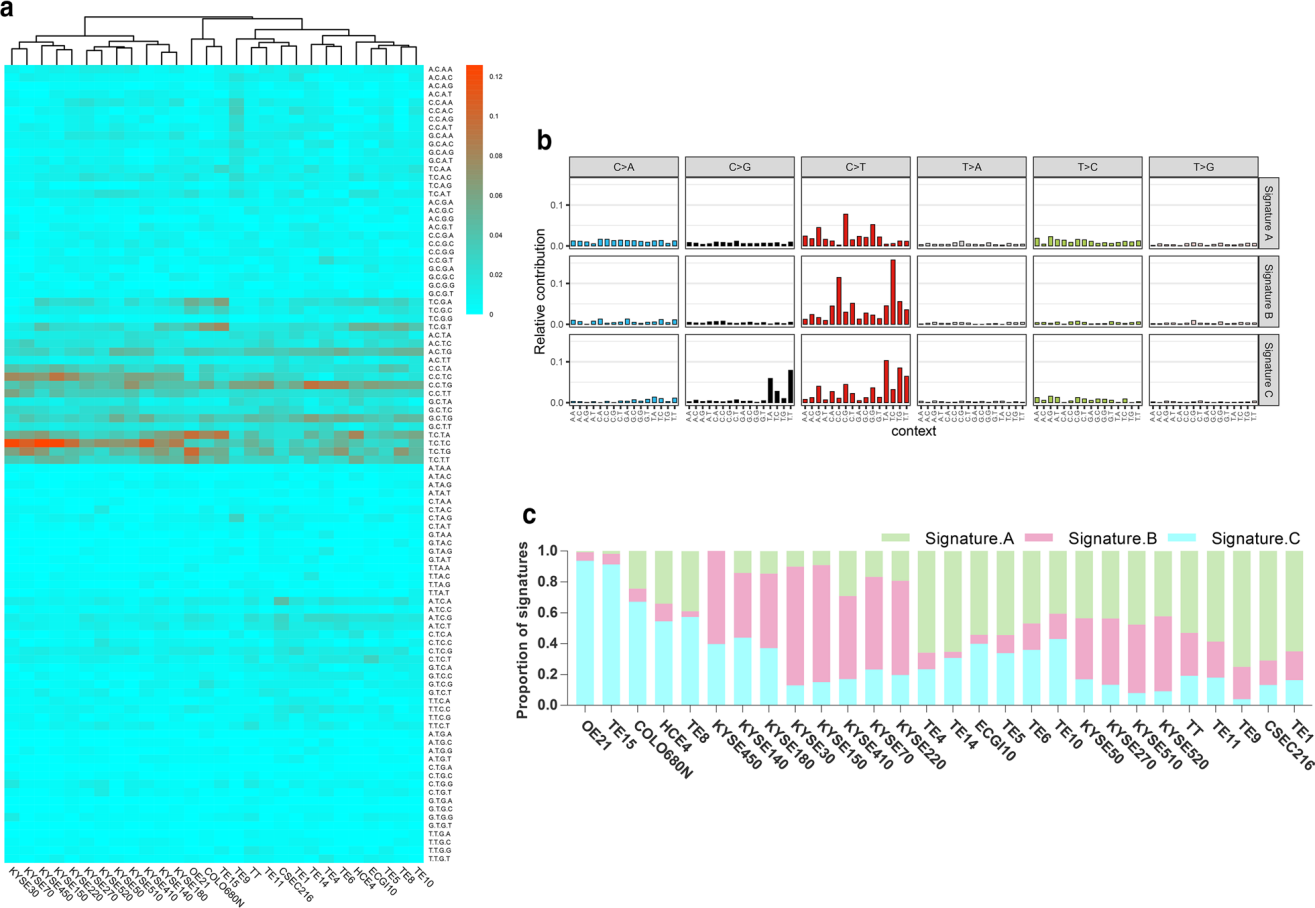
96 trinucleodide mutations spectrum and *de novo* mutational signatures derived from ESCC cell lines. **a** Characters of 96 trinucleodide mutations among ESCC cell lines. **b** 96 trinucleodide mutations spectrum of three *de novo* mutation signatures. **c** Optimal contribution of the three *de novo* signatures to each ESCC cell line’s mutation profile

After removing problematic cell lines, a total of 28 SNA and 24 CNA mutation data were downloaded from CCLE database (https://portals.broadinstitute.org/ccle/about). CNA and SNA data of cell line were shown in Additional file 7: Tables S6, Additional file 8: Tables S7. Firstly, the mutation matrix of each cell line with 96 SNA mutations at trinucleotide context were compared (Fig. 6a). The spectrum of 96 mutation context of 28 cell lines were shown in Additional file 9: Figure S2. Globally, high rate of C > T transition at TpCpX, CpCpX, ApCpG and C > G transversion at TpCpA/C/T trinucleotide were commonly found among cell lines. However, CSEC216 showed relatively lower mutation rate at the above mentioned sites, but uniquely possessed high mutation rate of T > C transition at ApTpA trinucleotide. De novo mutation signatures were extracted from ESCC cell lines by non-negative matrix factorization (NMF) algorithm in MutationalPatterns package. Three mutation signatures were extracted (Fig. 6b). Signature A was featured by high rate of C > T transition at XpCpG trinucleotide. After comparing with COSMIC Mutational Signatures, Signature A was similar to COSMIC Cancer Signature 1, which has been commonly found among different cancer types, also proposed to be the result of spontaneous deamination of 5-methylcytosine and has a relation with aging [18, 19]. Signature B had dominant C > T transition at CpCpC and TpCpC trinucleotide, after comparison with COSMIC database, Signature 11 was most similar. Signature 11 has been found in melanoma and glioblastoma, the mutational pattern may caused by treatments with the alkylating agent temozolomide [18]. Since temozolomide is not prevalently used to treat ESCC patient, the aetiology of this Signature B remained unclear. Signature C exhibited high rate of C > G and C > T transition transversion at TpCpX trinucleotide. When comparing with COSMIC database, Signature B was similar to Signature 2, which has been identified in variety cancer types, and possibly affected by the activity of APOBEC family of cytidine deaminases, especially APOBEC1/3A/3B [18, 20].

The contribution of Signature A/B/C to the mutational patterns of each cell line was compared. ESCC cell lines demonstrated diverse composition of mutation signatures. Signature A/B/C was dominant(proportion > 50%) in 32.1% (9 of 28)/21.4% (6 of 28)/17.9% (5 of 28) cell lines respectively. Extreme cases were found in OE21 and TE15 where Signature C take 93.7% and 91.3% proportion among the 3 signatures (Fig. 6c).

For ESCC cell lines researching on Cellosaurus, 224 hits plus 2 ESCC cell lines were retrieved, 12 of which were problematic cell lines because of cell line cross-contamination. Meanwhile, information of 14 immortalized human esophageal epithelial cell lines were collected from Celloasurus and published literatures. Information of those cell lines were shown in appendices.

**Fig. 7:**
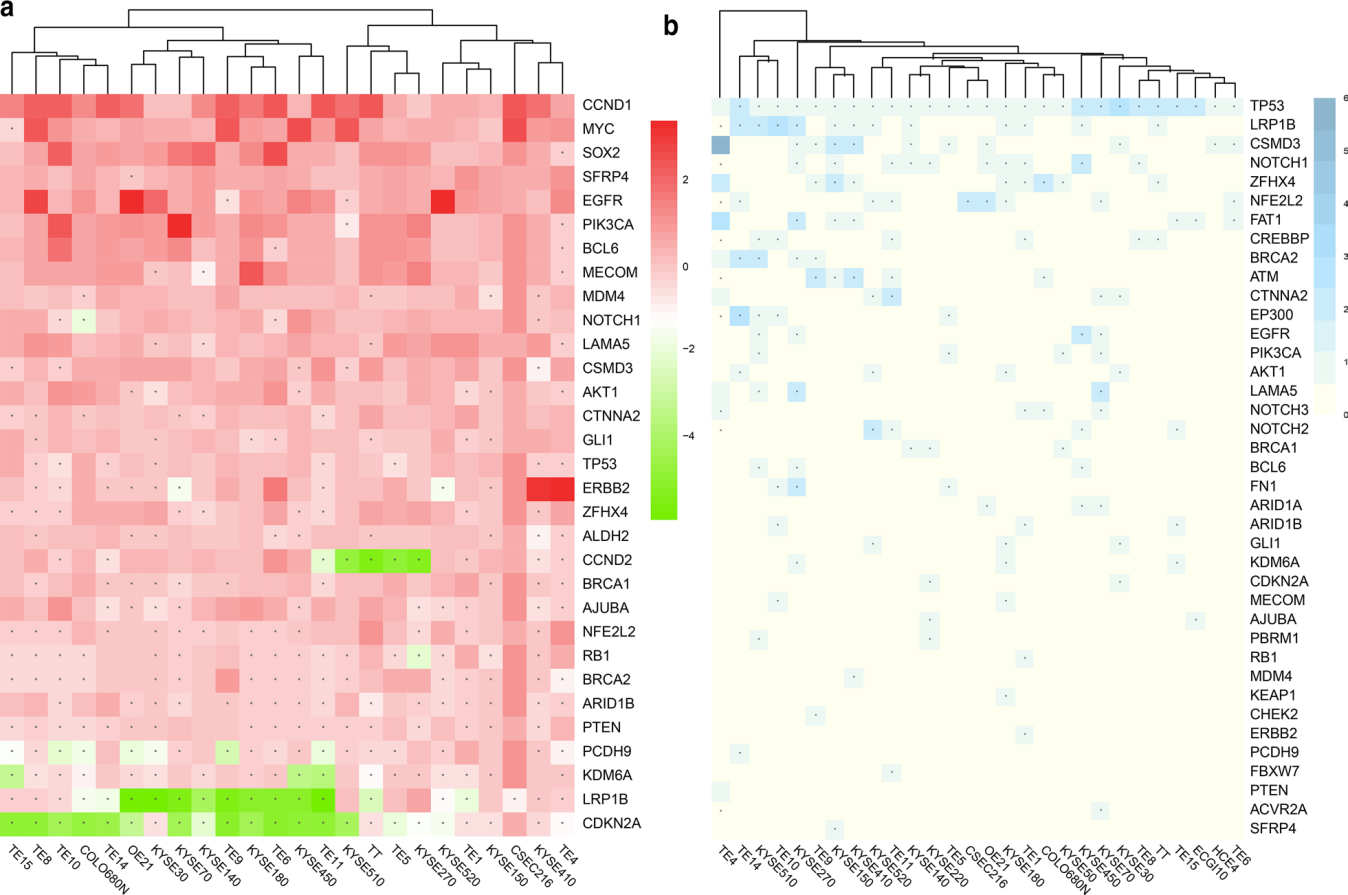
Mutations status of ESCC related genes in SNA and CNA profiles. **a** Copy number alterations mutation status of ESCC related genes among ESCC cell lines. Genes with copy number loss were labelled by asterisk. **b** single nucleotide alterations, mutation status of ESCC related genes among ESCC cell lines. Genes had mutations were labelled by asterisk

In order to investigate the mutation status of genes that have been reported being mutated in ESCC, genes that had somatic mutation at exonic or up/down stream region were selected from ESCC cell lines and mapped to 44 ESCC related genes. 39 genes were identified in SNA/INDEL data, TP53 undoubtedly was the most prevalent one, every cell line possessed 1 or 2 mutations in TP53, followed by LRP1B (42.9%), CSMD3 (39.3%), NOTCH1 (35.7%), ZFHX4 (32.143%), NFE2L2 (28.6%) (Fig. 7b). 31 genes were identified in CNA data, the most significantly altered genes were CCND1, which amplified in every cell line, followed by MYC, SOX2, SFRP4 (95.8%), EGFR, PIK3CA, BCL6 (91.7%). CDKN2A was found deleted in 95.8% cell lines, followed by LRP1B (83.3%), KDM6A, PCDH9 (75%), PTEN (70.8%), ARID1B, BRCA2, RB1, NFE2L2 (66.7%) (Fig. 7a).

## Discussion

Esophageal carcinoma is one of the most common and lethal malignancies in China and worldwide [2, 21]. Prognosis is poor and challenges remain in early diagnose and efficient treatment. The in vitro cell models together with human xenograft models may be useful in predicting the performance of cancer drugs and reveal mechanisms within tumorigenesis and metastasis [22].

Short tandem repeat profiling is the standard method for authenticating cell lines by using at least eight STR loci and the application of match criteria [23, 24]. By comparing 8 loci and AMEL with STR DATABASE in ATCC and DSMZ, several similar cell lines had been identified, however, none of these cell lines was cultured during the establishment of CSEC216. Therefore, this newly established ESCC cell line CSEC216 had been confirmed as unique and uncontaminated cell line.

Aneuploidy and chromosome aberrations in human solid tumors are hallmarks of gene deregulation and genome instability [25, 26]. There is a broad range of chromosomal abnormalities in solid tumors, including altered ploidy, gain or loss of individual chromosomes or portions thereof, and structural rearrangements. Previous studies and DSMZ online resources demonstrated that cell lines derived from ESCC harbored frequent chromosome number variation [27–30]. Gain of chromosome number was common with 1,3,7,10,11,20, and loss of chromosome number was pervasive on 2, 4, 5, 9, 13, 14, 15, 18, 19, 21, 22, Y. In this study, numerical abnormalities were basically in accordance with previous findings, only Chr 5 and Chr 11 were exceptions. Chromosome Y, albeit had not been detected in SKY analysis, but sex determine loci AMEL were identified in STR profiling and sequence of Chr Y were captured in WGS. Foregoing results suggested that the loss of Chr Y in karyotypic analysis might due to Chr Y had translocated with other chromosomes into small fragments that out of the resolution limit of SKY, while the nucleotide sequence were remain detectable in STR profiling and WGS.

Mutation process during carcinogenesis leave traces on the genome, which could be discriminated by different features, i.e. mutation type, transcriptional strand bias and sequence context. Mutation signatures are combined features of cataloged mutation types which are footprint from variety mutagenesis processes [6]. The de novo mutation Signature A and C that were extracted from ESCC cell lines, similar de novo mutation signatures were also found in other ESCC genomic studies [12, 17, 31, 32]. The similarity of de novo mutation signatures between ESCC tumors and tumor derived cell lines confirmed the consistency of the genomic context within ESCC and its in vitro disease models. The diversity of the compositions of signatures could let researchers choose those cell lines that best fit their needs.

CNA is one of major mutation in human cells. Normal human cells are diploid, but in tumor cells, there are copy number amplified or deleted regions in the genome [7]. Deletions of genome in some cancers could lead to inactivation of cancer suppressor genes like RB1, P16, PTEN, etc. While genome amplification could activate some cancer promoter genes, like MYC, ERBB2, EGFR, etc. Those genes have important roles in many pathways that could promote or suppress cellular behaviors [33]. Copy number gain at 3q, 5p, 8q, 12p, 20p, 20q and loss 3p, 4q, 4q, 5q, 9p, 10p, 13q, 18q, 19p, 21q in ESCC cancer cells have been reported in ESCC [34, 35]. Comparing with those already known regions, CSEC216 cells showed broader region of large scale CNAs especially copy number gain. Thousands of genes were located in CNA regions, mostly amplified. Including several above mentioned ESCC related genes.

We also investigated mutation status of ESCC related genes in ESCC cell lines. Genes that participate in cell cycle regulation, cell differentiation, oxidative damage, etc. were found to have SNA or CNA mutations in ESCC cell line. Large scale genomic data analysis had been pulled out by the TCGA [16]. Their results showed that ESCC could be classified into 3 molecular subgroups. Subgroup I was prevalent among Asian ESCC patient, featured by aberrances in NFE2L2 pathway and SOX2 amplification. Such alterations were found in most of ESCC cell lines, including CSEC216. Indicating that these cell lines had similar molecular profiles with original tumors.

## Conclusion

In summary, a novel and well characterized human ESCC cell line CSEC216 has been established. CSEC216 was derived from a 45-year old male ESCC patient from Chaoshan littoral, China. CSEC216 cells exhibit epithelial cell features and possess high proliferative activity, relatively low in vitro migration and invasion ability. CSEC216 exhibited hypotriploidy karyotype with complicated chromosomal aberrations. One somatic stop gain mutation and two somatic missense mutations were found in TP53 and NFE2L2 at exonic region respectively. MYC, CCND1, SOX2, PIK3CA, TP53, et al. were found gene copy number gained, while LRP1B was found copy number lost among selected ESCC related genes. Genomic background study of CSEC216 together with 28 ESCC cell lines demonstrated that those cellular disease models, to some extent, at least genetically, might be representable of ESCC. The current study could provide useful ESCC cell line information which would facilitate researchers for choosing appropriate cell model for their study.

## Supplementary information

**Additional file 1: Table S1.** ESCC cell line information from Cellosaurus and literatures.

**Additional file 2: Table S2.** Background information of CCLE ESCC cell lines.

**Additional file 3: Figure S1.** Report and electropherogram of STR profiling. A, STR profiling report of CSEC216 at P5. For what’s worth mentioning, CSEC216 was originally designated as ECST109, to distinguish from widely used ESCC cell line EC109, the name was changed as CSEC216. B, Electropherogram.

**Additional file 4: Table S3.** SNA and INDEL data of CSEC216.

**Additional file 5: Table S4.** CNA data of CSEC216.

**Additional file 6: Table S5.** List of 44 genes related with ESCC were selected from literatures and COSMIC database.

**Additional file 7: Table S6.** CNA data of 24 ESCC cell lines.

**Additional file 8: Table S7.** SNA data of 28 ESCC cell lines.

**Additional file 9: Figure S2.** SNA spectrum of ESCC cell lines. The spectrum of 96 mutation context of 28 ESCC cell lines.

## Abbreviations

EC: Esophageal cancer
ESCC: Esophageal squamous cell carcinoma
PDT: Population doubling time
IF: Immunofluorescence
SKY: Spectral karyotyping
STR: Short tandem repeats
WGS: Whole genome sequencing
SNA: Single nucleotide alteration
INDEL: Insertion and deletion
CNA: Copy number alteration
