# Supplementary figures and images for: Genomic characterization of a newly established esophageal squamous cell carcinoma cell line from China and published esophageal squamous cell carcinoma cell lines
Source: Cancer Cell Int. 2020 May 24;20:184. doi: 10.1186/s12935-020-01268-x (PMC7247234; doi:10.1186/s12935-020-01268-x)

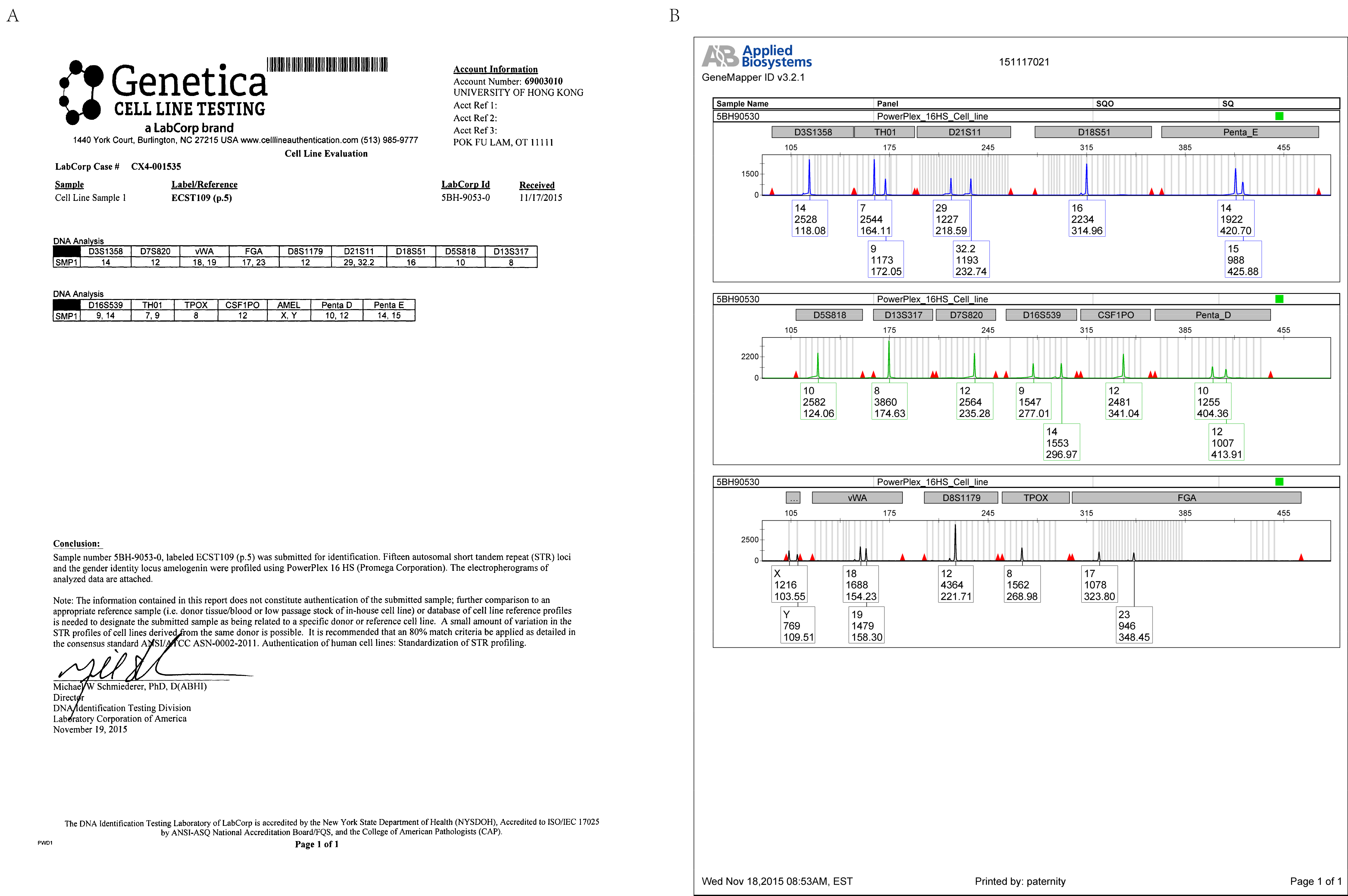

Supplement: Supplementary file 3 — Additional file 3: Figure S1. Report and electropherogram of STR profiling. A, STR profiling report of CSEC216 at P5. For what’s worth mentioning, CSEC216 was originally designated as ECST109, to distinguish from widely used ESCC cell line EC109, the name was changed as CSEC216. B, Electropherogram. [file 12935_2020_1268_MOESM3_ESM.tif]

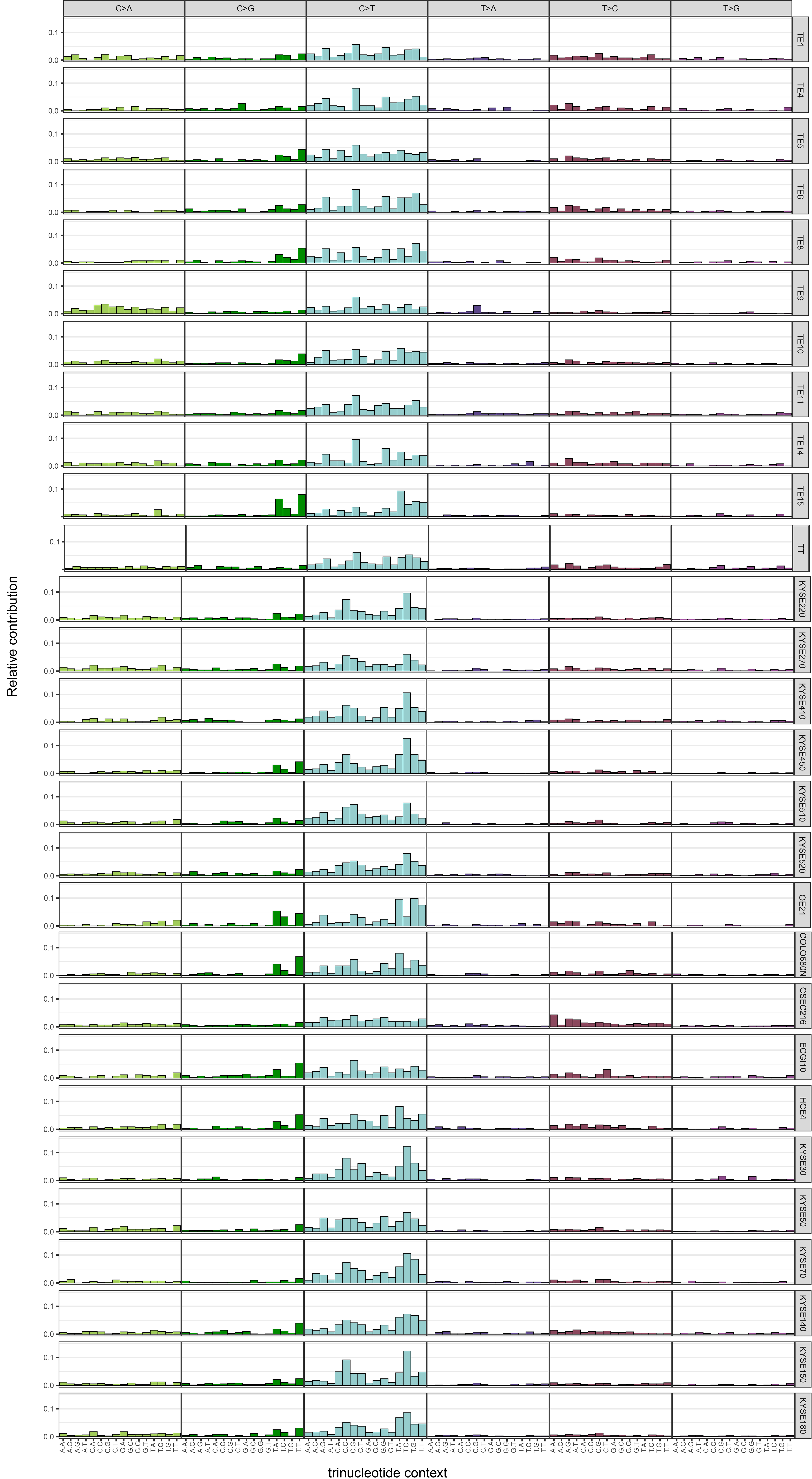

Supplement: Supplementary file 9 — Additional file 9: Figure S2. SNA spectrum of ESCC cell lines. The spectrum of 96 mutation context of 28 ESCC cell lines. [file 12935_2020_1268_MOESM9_ESM.tif]
